# Optical Behavior of Clear Thermoplastic Dental Materials in a Simulated Oral Environment

**DOI:** 10.3390/polym17040472

**Published:** 2025-02-11

**Authors:** Liliana Porojan, Flavia Roxana Bejan, Roxana Diana Vasiliu, Anamaria Matichescu

**Affiliations:** 1 Department of Dental Prostheses Technology (Dental Technology), Center for Advanced Technologies in Dental Prosthodontics, Faculty of Dental Medicine, “Victor Babeș” University of Medicine and Pharmacy Timișoara, Eftimie Murgu Sq. No. 2, 300041 Timișoara, Romania; sliliana@umft.ro (L.P.); roxana.vasiliu@umft.ro (R.D.V.); 2 Department of Preventive, Community Dentistry and Oral Health, Center for Advanced Technologies in Dental Prosthodontics, Faculty of Dental Medicine, “Victor Babeș” University of Medicine and Pharmacy Timișoara, Eftimie Murgu Sq. No. 2, 300041 Timișoara, Romania; matichescu.anamaria@umft.ro

**Keywords:** clear thermoplastic dental material, simulated oral environment, optical properties, color, whiteness

## Abstract

(1) Background: The intra-oral behavior of clear thermoplastic dental materials can be influenced by various intrinsic and extrinsic factors. Aim: The purpose of this in vitro study was to evaluate the optical properties, color changes and whiteness variations of four thermoplastic polymers used for dental appliances, in a simulated oral environment. (2) Methods: Customized thermoformed specimens of four PETG thermoplastics were selected and investigated in this study: Leone [L], Duran [D], Erkodur [E] and Crystal [C]. The thermoplastic samples were divided into three groups related to pH values (neutral, acidic and basic). A period of 14 days was simulated. Five stages resulted: I. dessicated specimens; II. hydrated in artificial saliva; III. subsequent desiccated; IV. artificial aged; V. further dessicated. Optical CIE L*a*b* coordinates were determined and optical properties, like TP (translucency), OP (opalescence) values, color differences ΔE-NBS, white indexes in dentistry WI_D_ and white index differences ΔWI_D_ were calculated for all stages of the study, for each group of the materials. Statistical analyses were performed. (3) Results: Optical properties of PETG clear thermoplastic materials, like TP and OP, increase in a simulated oral environment and the changes become significant after artificial aging. Related to pH values, the optical behavior between the materials is significantly different. During artificial aging, the tested materials behave significantly differently in terms of optical properties. (4) Conclusions: After the simulated period of 14 days, TP and OP values increase, with a migration of the color towards red and yellow. Color changes in some cases even reach the level of extremely marked. Whiteness increases, and the differences are mostly perceptible, but partially exceed the limit of acceptability.

## 1. Introduction

The use of clear thermoformed plastic materials for the fabrication of custom-made dental appliances, like clear aligners, retainers, mouthguards and periodontal splints, is a common feature, related to their excellent properties [1,2,3]. Clear aligners are discreet and can be removed, offering more comfort for the patients. They are changed sequentially every one to two weeks, according to the treatment plan. They combine strength and flexibility [4]. The classical method of vacuum thermoforming using a thermoplastic material molding on physical models is widely used, beside the last developed 3D printing [5,6,7,8]. Studies show that thermoformed materials generally still have lower water sorption, water solubility, and surface roughness, higher transparency and color stability than 3D-printed resins [9].

Thermoplastic polymers can be divided into amorphous and semicrystalline polymers, related to their molecular structure. Amorphous materials are characterized by irregular structures, and semicrystalline polymers by combined uniform (crystalline) and irregular areas (amorphous). The crystalline part confers hardness and rigidity. Amorphous polymers are more flexible, soft and transparent, which allows light to pass through them easily and exhibit low shrinkage; they are impact resistant and easier to process. Semicrystalline polymers, with their unique structure, combine the properties of both crystalline and amorphous materials. They are rigid, strong, resistant to wear, opaque or translucent [1,6,10]. The intra-oral behavior of these polymers can be influenced by various factors, which depend, on the one hand, on the properties of the materials and, on the other hand, on the oral environment [1,4].

Polyethylene terephthalate glycol (PETG) is a non-crystalline, amorphous copolyester, with excellent mechanical and optical properties, resistant against chemical environmental changes and suitable for clear thermoplastic devices [11].

For this study, four common commercial PETG materials, frequently used in clinical practice and with almost similar composition, were chosen for comparison.

Aesthetics plays an important role for clear appliances, and the optical properties are expected to undergo no changes during the period of use. It was shown that the optical stability is affected by the consumption of colored beverages, because half of the patients did not remove the appliances for drinking, saliva and mouthwashes. Thermoplastics can undergo irreversible hydrolysis, potentially compromising polymer integrity [12,13,14].

The influence of colored beverages could be related to pH values and temperature variations to simulate the oral environment. Different studies have evaluated the color change of clear aligners in vitro and under ex vivo conditions and have shown that color changes were minimal after immersion in red wine, but significant after immersion in coffee solution, with minimal overall discoloration [15].

Previous studies comparing PETG and polyurethane aligners have highlighted the high level of transparency of PETG in accordance with their degree of crystallinity. Independent of the staining solution (artificial saliva, wine, coffee), PETG specimens showed perceptible color changes already after 7 days and after 14 days; UV–visible spectrophotometry analyses revealed an increase (up to 30%) in the absorbance value along all wavelengths—after immersion in coffee and wine—indicating a loss of transparency due to interaction with the staining agents [16].

Dehydration may induce color alterations [2,3,4,5,6], increasing the lightness. The decrease in translucency is justified by the replacement of water with air, altering light dispersion, increasing the reflection and increasing the luminosity [17,18,19].

Because thermoplastic devices are subject to long-term exposure to an intra-oral environment with temperature variations, the materials should be resistant to hydrothermal degradation [9]. Specific conditions of the oral environment, such as biofilm formation, salivary enzymes, bacterial environment, temperature and pH, can negatively affect the maintenance of aligner transparency during treatment. The range of intra-oral temperatures has been observed to vary between 5.6 °C and 58.5 °C, which are the temperatures used in the accelerated aging method of dental materials, by thermal cycling. However, patients should remove clear retainers during consumption of beverages and food, so that the materials are not subjected to extreme temperature fluctuations. In conclusion, the color change of aligners can be determined by the quality of the surface of the material, allowing the adsorption of liquids or the penetration of pigments into the polymer, depending on the diet of each individual, and also, on the variation in temperature, the characteristics of saliva (pH, enzymes), and the form and frequency of hygiene [20].

For the study of the optical properties in dental research, the CIE L*a*b* color space proposed by the International Commission on Illumination (CIE) is widely used. For color difference calculations, the CIE L*a*b* total color difference (ΔE^∗^) is used. Aesthetics plays an important role for thermoplastic appliances; therefore, their optical properties are important to be preserved [21,22].

Color stability of clear appliances can be evaluated by analyzing parameters such as translucency and opalescence. The challenge of performing in vitro studies in order to evaluate their optical characteristics requires a careful control of the intrinsic and extrinsic factors which simulate the intra-oral behavior [23].

Preserving translucency and opalescence are key factors for aesthetic results, wearing clear appliances. Translucency expresses the amount of light transmitted from the substrate, and it is the state between opacity and transparency. Opalescence confers the bluish-white appearance in reflected light and an orange-brown appearance in transmitted light [24,25,26,27,28].

Additionally, to color evaluations, whiteness values are important for research. Lately, a whiteness index especially designed for dental applications (WI_D_) was proposed, based on the CIE L*a*b* color space. It is calculated based on the three CIE L*a*b* color coordinates. High values of WI_D_ are associated with high whiteness and lower values with decreased whiteness. This WI_D_ index is highly correlated to the visual perception registration [17,21,28,29,30,31,32].

The purpose of this in vitro study was to evaluate the optical properties, color changes and whiteness variations of four thermoplastic polymers used for dental appliances, in a simulated oral environment (hydration in artificial saliva with different pH values, hydrothermal degradation, desiccation).

The first null hypothesis was that the optical properties of PETG samples are influenced by immersion in artificial saliva and pH values. The second null hypothesis was that the optical properties of PETG samples are influenced by hydrothermal degradation. The third null hypothesis was that various commercial PETG materials behave similarly in terms of optical properties. The fourth null hypothesis is that color and whiteness changes are above perceptibility thresholds.

## 2. Materials and Methods

### 2.1. Sample Preparation

Customized specimens of four types of PETG thermoplastics were selected and investigated in this study: Leone (Leone SpA, Firenze, Italy)—noted L, Duran (Scheu-Dental GmbH, Iserlohn, Germany)—noted D, Erkodur (Erkodent, Pfalzgrafenweiler, Germany)—noted E, Crystal (Bio Art Dental Equipment, Sao Carlos, Brazil)—noted C. For thermoforming, calibrated thermoplastic sheets (1 mm thick) and a pressure molding device, MINISTAR S (Scheu-Dental, Iserlohn, Germany), were used. A gypsum mold was obtained, with surfaces of L = 40 mm × W = 10 mm, positioned at an angle of 45°. Each sheet was heated (220 °C/30 s) to malleability, then vacuumed (4 bar) and pressed over this mold, positioned in the pallet container. After cooling, the thermoformed sheet was removed, and square samples (10 × 10 mm) were cut. After thermoforming, the final thickness varied, depending on the material. Thirty specimens of each thermoplastic material (a total of 120 pieces) were prepared and analyzed. All samples were first stored in an airtight glass container, with silica gel, placed in an incubator at 37 °C until desiccation and assessed as control specimens (Dc, Lc, Ec, Cc). In the second stage of the experiment, the thermoplastic samples were divided into three groups and stored for 14 days in glass recipients containing modified Fusayama artificial saliva solution, containing the following [g/L]: Urea 1.0, CaCl_2_·2H_2_O 0.795, NaH_2_PO_4_·H_2_O 0.690, NaCl 0.4, KCl 0.4, KSCN 0.3, Na_2_S·9H_2_O 0.005. All reagents were used without further purification; the constituents were dissolved in bi-distilled water, and the pH level was set to the desired values. Ten specimens of each polymer were deposited in saliva with neutral pH_0_ = 6.7 (group L_0_, D_0_, E_0_, C_0_), with acidic pH− = 4.3 (group L−, D−, E−, C−) and basic pH+ = 8.3 (group L+, D+, E+, C+), and analyzed.

In the third stage of the study, the three groups of specimens were re-dried in glass containers containing silica gel and stored in a desiccator at 37 °C for 7 days.

In the fourth stage of the study, samples were subjected to hydrothermal aging by thermocycling: a total of 400 cycles, in cold (5 °C) and warm (55 °C) water bath. Each cycle lasted 80 s (30 s dwell time and 10 s transfer time) and was performed to simulate 14 days of intra-oral use.

In the fifth stage of the investigation, all plates were re-dried in glass containers containing silica gel and stored in a desiccator at 37 °C for 7 days.

### 2.2. Optical and Color Changes Measurements

Optical CIE L*a*b* coordinates were determined and optical properties, like TP (translucency), OP (opalescence) values, color differences ΔE-NBS, white indexes in dentistry WI_D_ and white index differences ΔWI_D_ were calculated for all stages of the study, for each group of the materials. The recording of optical parameters was performed using a spectrophotometer, under D65 illuminate, using Easyshade IV (Vita Zahnfabrik, Bad Säckingen, Germany), which was calibrated before each evaluation. It is a clinical instrument that only works in “tooth mode” [33].

The measurements were accomplished on two different backgrounds, white (w) and black (b), of the grey card WhiBal G7 (White Balance Pocket Card) with the probe tip positioned 90° to the sample surface. The measurements were carried out by the same operator in five randomly selected areas of one surface. L* represents the lightness coordinate (perfect black: L* = 0, perfect white: L* = 100), a* represents the chromatic coordinate in the red (positive value) or green (negative value) axis, while b* is the chromatic coordinate in the yellow (positive value) or blue (negative value) axis [34].

TP (translucency) and OP (opalescence) values were calculated according to the following equations:

TP = [(L*_b_ − L*_w_)^2^ + (a*_b_ − a*_w_)^2^ + (b*_b_ − b*_w_)^2^]^1/2^ (1)

Translucency (TP) values can range from 0 (completely opaque) to 100 (full transparent).

OP = [(a*_b_ − a*_w_)^2^ +(b*_b_ − b*_w_)^2^]^1/2^ (2)

OP values determine the difference in red–green and yellow–blue chromatic coordinates between reflected and transmitted light.

The color difference between two stages—ΔE* (total color change value)—was obtained using the following formula:

ΔE* = [(ΔL*) ^2^ + (Δa*) ^2^ + (Δb*) ^2^] ^1⁄2^ (3)

Recordings were made for each group, for each stage.

To relate the color change to a clinical standard, ΔE* was subsequently converted into NBS units (NBS = National Bureau of Standards) using the following formula [25,35]:

NBS = ΔE* × 0.92(4)

The levels of color change, expressed in NBS units, are as follows: 0.0–0.5 extremely slight change, 0.5–1.5 slight change, 1.5–3.0 perceivable, 3.0–6.0 marked change, 6.0–12.0 extremely marked change, 12.0 or more—change to another color.

The whiteness index in dentistry (WI_D_) and white index differences ΔWI_D_ were calculated in order to quantify the degree of whiteness of the materials, according to the following equations [29].

WI_D_ = 0.511 × L* − 2.324 × a* − 1.1 × b* (5)

ΔWI_D_ = WI_D_2 − WI_D_1 (6)

To obtain WI_D_ differences, the mathematical difference between the values registered in one stage and those in the control group, for the same material, were calculated.

Thresholds used for color evaluations are important quality control tools. The perceptibility thresholds (WPT) and acceptability thresholds (WAT) were established at 0.72 and 2.60 WID units [36].

### 2.3. Statistical Analysis

IBM SPSS Statistics (IBM, New York, NY, USA) and JASP (v.16.2, University of Amsterdam, Amsterdam, The Netherlands) software were used to achieve the statistical analysis. For all materials and surfaces studied in the five stages, the mean values for the color parameters and the standard deviations were calculated.

Initially, using descriptive statistics, dispersion parameters and central tendency were calculated. To establish the distribution of the data, the Shapiro–Wilk test was applied. In some cases, they were normally distributed, and in others, they were not normally distributed; therefore, in order to have a complete perspective, parametric and non-parametric tests were applied.

The comparison of samples immersed in artificial saliva with different pH values—for each material, within a stage—were performed using the one-way ANOVA (normal)/Kruskal Wallis (not normal distribution) tests and subsequently, post hoc Tukey tests for multiple comparisons for pairwise combinations. Student *t*-test unpaired (normal)/Mann–Whitney (not normal distribution) tests were applied to compare different materials immersed in the same environment, in one stage. Student *t*-test paired (normal)/Wilcoxon signed-rank (not normal distribution) tests were used for two-time moments, comparisons between two stages (before and after) for the same material.

Pearson correlation and regression analyses were applied to assess the interdependence between the recorded variables and the optical parameters (TP, OP) at different stages.

The signification of the Pearson coefficient (r) was related to “very weak” [0–0.2], “weak” [0.2–0.4], “moderate” [0.4–0.6], “strong” [0.6–0.8]”, and “very strong” [0.8–1.0].

The coefficient of determinations (r^2^) denotes the proportion (%) of the total variation of the dependent variable (L*, a* or b*) that can be defined by the variation of the independent variable (material, TP, OP). For all, a value of *p* < 0.05 was considered significant. The Bonferroni correction was applied additionally, which significantly reduced the strength of the underlying tests; the original α level (0.05) was adjusted to the new α.

## 3. Results

**Figure 1:**
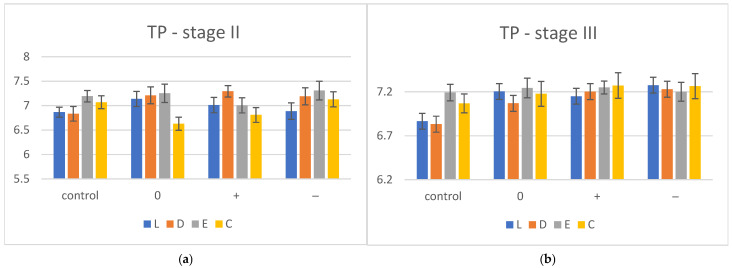
Translucency (TP) evolution for hydration (**a**) and desiccation conditions (**b**), (0) = neutral artificial saliva, (−) = acidic artificial saliva, (+) = basic artificial saliva, L = Leone, D = Duran, C = Crystal, E = Erkodur.

The coordinates L*, a* and b* were recorded on a white background and the resulting values for TP and OP for each material in the control group (stage I), the immersed group (stage II) in artificial saliva with different pH values: 6.7 = neutral (0), 8.3 = basic (+), 4.3 = acid (−) and then re-dried (stage III) were calculated. The mean TP values with standard deviation (SD) for dried control (stage I), hydrated (stage II) and reconditioned (stage III) groups are summarized in Table 1 and displayed graphically in Figure 1a,b.

After immersion—stage II—an increasing trend in TP values was observed, except for C (TP decreased in all media). In the control group, the most translucent material was E and the values remained the highest even after hydration (pH 0, +, −). The one-way ANOVA (α = 0.05), post hoc Tukey (pairwise comparison) statistical test and Bonferroni correction showed significant differences between immersed samples of a material, for E (E+ and E−, E0 and E+) and C (C0 and C−, C+ and C−).

After reconstruction by desiccation—stage III—the TP values increased for all materials in all environments. The translucency of E (0, +, −) is not affected by re-drying, the values being almost similar to those in stage II, but this material remained the most translucent compared to the other groups. Insignificant differences (*p* > 0.05) were reported regarding pH values when the one-way ANOVA test and Tukey post hoc test were applied, for all four materials.

The Student *t*-test (unpaired) was applied to compare the translucency of different materials in the same pH media (0, +, −) and reported significant differences (*p* < 0.05) in the control and hydrated groups (especially in the + group), and insignificant differences in the desiccated ones (Table 2).

The Student *t*-test (paired) and Bonferroni correction were applied to compare the TP values among control (I) and one-stage (II or III) samples for the same material. The statistical test on TP values for stage I–II revealed significant differences between D (all), Cc and C0 and for stage I–III, between Dc and D+, Dc and D−, insignificant differences being shown between E (all), Lc and L+, Cc and C− for both stages (I–II and I–III) (Table 3).

Pearson correlation (r) and regression tests (r^2^) were performed to assess the relation between the optical coordinates (L*, a*, b*) and optical parameter TP for Lc and L0, D (all), Cc and C0, (after immersion), and between Lc and L0, Dc and D+, Cc and C (after desiccation) (Table 4).

**Figure 2:**
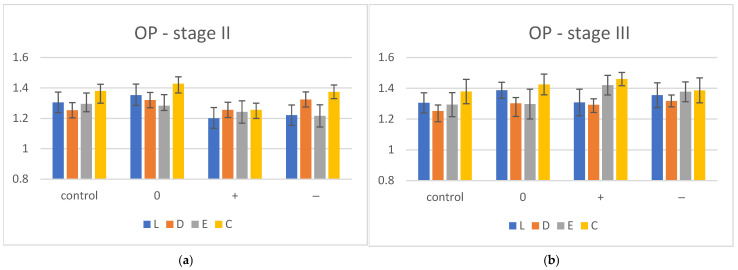
Opalescence (OP) evolution for hydration (**a**) and desiccation conditions (**b**), (0) = neutral, (−) = acidic, (+) = basic, C = Crystal, E = Erkodur, L = Leone, D = Duran.

After immersion (stage II), an increase in OP values was observed for D and a decreasing trend for E in all media. The opalescence decreased for L and C in (−, +) environments. In the control group, the most opalescent material was C and the values remained the highest even after hydration (pH 0, +, −). The mean OP values with standard deviation (SD) for dried control (stage I), hydrated (stage II) and reconditioned (stage III) groups are summarized in Table 5 and displayed graphically in Figure 2a,b.

The one-way ANOVA (α = 0.05), post hoc Tukey (pairwise comparison) statistical test and Bonferroni correction reported significant differences between immersed samples of a material for L (L0 and L+, L0 and L−), E (E+ and E−) and C (C0 and C+, C+ and C−).

After being re-dried (stage III), an increase in OP values for all three materials in all types (0, −, +) of media was reported. The opalescence of D (0, +, −) is not affected by re-drying, the values being almost similar to those in stage II, and C remained the material with the highest OP values compared to the other groups. Significant differences (*p* > 0.05) were reported regarding pH values when the one-way ANOVA test, Tukey post hoc test and Bonferroni correction were applied for E (E0 and E+, E0 and E−) and C (C0 and C−, C+ and C−).

The Student *t*-test (unpaired) was performed to compare the opalescence of different materials immersed in the same environment (0, + or −) and reported significant differences (*p* < 0.05) in the hydrated and desiccated groups (Table 6).

The statistical Student *t*-test (paired) and Bonferroni correction were applied to compare the OP values among control (I) and one-stage (II or III) samples. Significant differences were shown between L (all) for both stages, and E (Ec and E+) for both stages (I, II and I–III) (Table 7).

Pearson correlation (r) and regression tests (r^2^) were performed to assess the relation between the optical coordinates (L*, a*, b*) and optical parameter OP for L (Lc and L−) for I–II stages, L (all) for I–III stages, and E (Ec and E+) and C (Cc and C+) for both stages (I–II and I–III)—Table 8.

According to the NBS system, after immersion and desiccation, the levels of color change were between extremely slight to extremely marked changes (Table 9).

**Figure 3:**
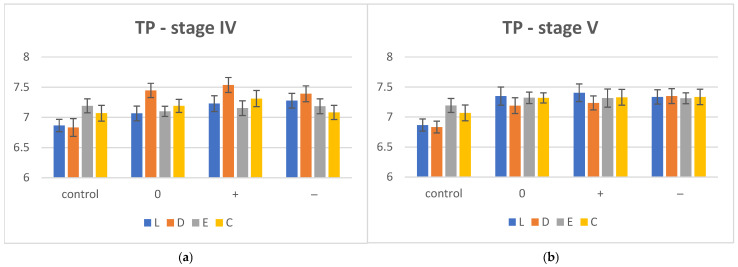
Translucency (TP) evolution for hydrothermal aging (**a**) and desiccation conditions (**b**), (0) = neutral, (−) = acidic, (+) = basic, C = Crystal, E = Erkodur, D = Duran, L = Leone.

The mean TP values with standard deviation (SD) for dried control (stage I), hydrothermal degradation (stage IV) and reconditioned (stage V) groups are summarized in Table 10 and displayed graphically in Figure 3a,b.

After hydrothermal aging by thermocycling—stage IV—a trend in increasing TP values, except for E (TP decreased in all media), was observed. Compared to the other materials, for D, the increase in translucency is more evident in all environments (pH 0, +, −). The one-way ANOVA (α = 0.05), post hoc Tukey (pairwise comparison) statistical test and Bonferroni correction showed significant differences between all samples of materials D (D+ and D−), E (E0 and E−) and C (C+ and C−).

After re-drying—stage V—the TP values increased for all materials in all environments. The translucency for L is the highest, and for E and C−, the values are almost similar in all three environments (0, +, −). Significant differences (*p* < 0.05) were reported regarding pH values when the one-way ANOVA test and Tukey post hoc test were applied for L (L0 and L−, L+ and L−) and C (C0 and C+, C0 and C−).

The Student *t*-test (unpaired) was applied to compare the translucency of different materials in the same pH media (0, +, −) and reported significant differences (*p* < 0.05) in almost all groups (Table 11).

The Student *t*-test (paired) and Bonferroni correction were performed to compare the TP values among control (I) and one-stage (IV or V) groups for the same material. The statistical test revealed significant differences between L (all) and D (all) for both situations (I–IV and I–V) (Table 12).

Pearson correlation (r) and regression tests (r^2^) were performed to assess the relation between the optical coordinates (L*, a*, b*) and optical parameter TP for Lc and L−, D (all) after immersion stage and L (all) and D (all) after desiccation (Table 13).

**Figure 4:**
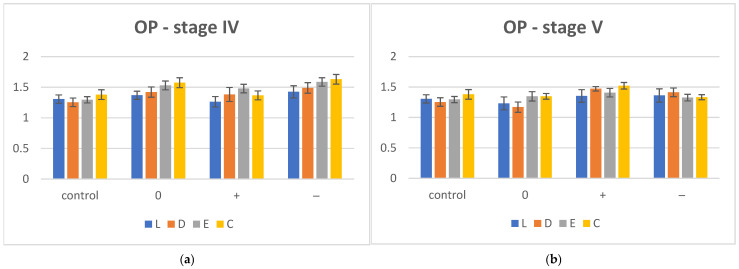
Opacity (OP) evolution for hydrothermal aging (**a**) and desiccation conditions (**b**), (0) = neutral, (−) = acidic, (+) = basic, C = Crystal, E = Erkodur, D = Duran, L = Leone.

The mean OP values with standard deviation (SD) for aged (stage IV) and reconditioned (stage V) groups are summarized in Table 14 and displayed graphically in Figure 4a,b.

After hydrothermal aging by thermocycling (stage IV), an increase in OP values was observed for all groups in the three pH environments: the most for C and the least for L. After reconstruction by desiccation, a decrease in OP values for all materials in neutral artificial saliva (pH = 0) was reported. In environments with pH+ and pH−, the values of opalescence increased.

The one-way ANOVA (α = 0.05), post hoc Tukey (pairwise comparison) statistical test and Bonferroni correction reported significant differences between almost pairwise in all three media (0, +, −), except for D (all) and L (L0 and L−, C (C0 and C−) in stage IV and for L (L0 and L+, L+ and L−), D (D+ and D−) and E (E0 and E+, E0 and E−).

The Student *t*-test (unpaired) was performed to compare the opalescence of different materials in the same pH media (0, +, −) and reported significant differences (*p* < 0.05) in almost all groups (Table 15).

Pearson correlation (r) and regression tests (r^2^) were applied to evaluate the relation between the optical coordinates (L*, a*, b*) and optical parameter OP for L (all) and D (all) in both stages, and for E (all) and C (all) in stage IV—Table 16.

According to the NBS system, after thermal aging and desiccation, the levels of color change were between slight changes to extremely marked changes (Table 17).

The whiteness index in dentistry (WI_D_) was calculated for the control group and for the samples subjected to hydration (stage II), desiccation (stage III), respectively, for those thermocycled (stage IV) and then re-dried (stage V) (Table 18).

ΔWI_D_ differences were calculated between the values obtained in different stages and the control group, for the same material, in the three immersion media (0, +, −) (Table 19).

## 4. Discussions

It was demonstrated by different studies that the optical properties, hardness and thickness of thermoplastic materials change during the thermoforming process; therefore, the investigations were oriented towards samples achieved after conventional processing, following the manufacturers’ instructions [37,38].

The optical behavior of the PETG thermoformed materials were investigated first in artificial saliva with different pH values. Water molecules from artificial saliva can bind and penetrate the surface of the material, allowing absorption of pigments and modifying the structure of the material.

PETG aligners are maintained in the oral cavity for approximately 14 days, then replaced with others until the treatment is completed. During this period, the material is subjected to the conditions of the oral environment, with a gradual deterioration of its optical and mechanical properties. The long-term optical stability over longer periods of time may be associated with the PETG retainers.

It has been observed that PETG materials do not undergo significant changes in terms of transparency after one day of aging [13]. A study revealed a visible staining capacity of clear aligners, but minimal differences in discoloration between PETG brands, after immersing samples in distilled water and beverages with different pH (orange juice, Pepsi, milk, black tea, coffee) for 7 days [39]. Another study compared the color changes between two transparent alignment systems (polyethylene terephthalate glycol and copolyester + thermoplastic elastomer) after immersion in various beverages for 14 days. The copolyester-based system is more prone to staining than PETG in coffee, cola and red wine solutions. The color parameters ΔL*, Δa*, Δb* and ΔE were affected by immersion in coffee solution for both materials [40]. Due to prolonged exposure to acidic beverages, these thermoplastic materials can suffer surface wear, becoming susceptible to discoloration [41]. Additionally, the temperature of beverages can cause thermal contraction or expansion of the material, increasing its susceptibility to discoloration and damage over time [42].

The increase in the surface roughness may also induce higher pigmentation and altering of the optical properties, as irregularities can retain coloring agents and impurities from beverages and foods [43,44,45,46,47]. When comparing several PETG materials, including Duran (Scheu-Dental GmbH, Iserlohn, Germany), low values of surface roughness resulted in a high degree of light transmission (>70%), but the 3D-printed materials with the highest surface roughness were clearly inferior, allowing for lower visible light transmission (<50%), appearing opaque [9].

The moisture oral environment influences the ability of the materials to maintain their properties during intra-oral use [9,46,47]. Because the degree of crystallinity is inversely related to transparency, these amorphous materials are expected to have excellent optical properties [48]. The sorption and water solubility values of a polymer are indicators of its resistance to mechanical and physical degradation [49]. Other studies that analyzed different thermoplastic materials reported that polyethylene terephthalate glycol and copolyester-based materials do not suffer significant changes in transparency after immersion in beverages, compared to polyurethanes, which can exhibit oxidative degradation, and have a higher absorption capacity and a rougher surface, all of which have an influence on enhancing coloration [50,51]. The commercial system Invisalign was invariably affected after 7 days of intra-oral use, and for the MaxDent CA digital system, it is indicated to replace the aligners every 10 days [52,53].

Regarding polymer aging, similar findings have been reported for resin cements [54], composite resins, as well as after 14-day in vitro or 6-month in vivo analyses on amorphous PETG leading to embrittlement and changes in mechanical and optical properties [55,56].

The CIE Lab color system recommends the use of L*a*b values. These values are closer to human perception, which is why they were chosen for this study. The use of a spectrophotometer and the CIE color system to measure color change allowed for a more quantitative and accurate assessment than the visual one. The threshold at which discoloration is visible to the naked eye is defined by a discoloration value ΔE ≥ 3.3. ΔE values in the range between 1 and 3.3 can be detected by some observers, while ΔE values < 1 are considered undetectable even by a trained eye [57].

A comprehensive review has been published that recommends a way to calculate thresholds for perceptibility and acceptability based on the samples and observers involved. Following evaluations of the literature, ΔE00 values of 1.1 and 2.8 (the lowest values reported) were considered as thresholds for perceptibility and acceptability, respectively. These may provide results that would have practical clinical applications [58].

One study reported significant differences between the threshold of perceptibility and acceptability, noting that these values differed between laypeople and dentists, who demonstrated better discrimination ability, reflected by lower values for both [36].

In other studies on color discrimination, laypeople were found to be less discriminating than dentists [59,60].

TP values increased for all materials in all environments after immersion in artificial saliva and re-drying, with significant differences found for all materials, in part of the environments (Dc/D+, Dc/D−). In the intermediate stage, during hydration, C material behaves differently, with a decrease in translucency. This can be related to a different structure, even if all belong to the same category, of PETG polymers. Insignificant differences (*p* > 0.05) related to TP were reported regarding pH values.

Pearson’s correlations and regression tests revealed strong and very strong positive correlations between TP and L* and strong negative between TP and a*, and only one strong positive between L and b*. It can be concluded that the increase in translucency is based on an increase in lightness, with a migration of the color towards green and yellow.

An increase in OP values for all three materials in all types (0, −, +) of media was reported after immersion in artificial saliva and re-drying, with significant differences between Lc/L+, Lc/L−, Lc/L0, Ec/E+.

Significant differences (*p* > 0.05) were reported regarding pH values for E (E0 and E+, E0 and E−) and C (C0 and C−, C+ and C−).

Pearson’s correlations and regression tests revealed strong and very strong positive correlations between OP and a*, b*, reflecting a migration towards red and yellow, with the increase in the opalescence.

Studies have shown that due to prolonged contact with saliva and especially with acidic beverages, thermoplastic materials can suffer surface wear, becoming susceptible to discoloration. The diffusion of water into the organic matrix can cause an increase in weight and/or volume due to hygroscopic expansion. Water absorption leads to hydrolytic degradation, having a negative impact on mechanical and aesthetic properties. Temperature variations can induce contraction or thermal expansion of these materials, further increasing their susceptibility to discoloration and deterioration [20,40,41,42].

The first null hypothesis that the optical properties of PETG samples are influenced by immersion in artificial saliva and pH values is only partially accepted.

After hydrothermal aging by thermocycling and re-drying, TP values increased for all materials in all environments. Significant differences (*p* < 0.05) were reported regarding pH values for L (L0 and L−, L+ and L−) and C (C0 and C+, C0 and C−).

Pearson’s correlations and regression tests revealed very strong positive correlations between TP and L*, and strong between TP and a* and b*. It can be concluded that the increase in translucency is based on an increase in lightness, with a migration towards red and yellow.

A statistical test reported significant differences for OP between almost pairwise in all three media (0, +, −) in desiccated thermocycled samples.

Pearson’s correlations and regression tests revealed strong and very strong positive correlations between OP and a*, b*, reflecting a migration towards red and yellow, with the increase in the opalescence.

The second null hypothesis that the optical properties are influenced by hydrothermal degradation is accepted.

Insignificant differences of TP values were found between different materials in the same pH media (0, +, −) in the re-dried stage, after saliva immersion. Tests performed to compare the opalescence of different materials immersed in the same environment (0, + or −) reported significant differences (*p* < 0.05) in the desiccated groups after saliva immersion. Translucency values of different materials in the same pH media (0, +, −) reported significant differences after thermocycling. Statistical tests performed to compare the opalescence of different materials after thermocycling, in the same pH media (0, +, −), reported significant differences (*p* < 0.05) in almost all groups.

The third null hypothesis that various commercial PETG materials behave similarly in terms of optical properties is partially accepted.

After saliva immersion and desiccation, the levels of color change were between extremely slight to extremely marked changes, with significant differences for Lc/L0, Lc/L+, Lc/L−, Dc/D0, Dc/D+, Dc/D−, Cc/C+. According to the NBS system, after thermal aging and desiccation, the levels of color change were between slight changes to extremely marked changes.

After 14 days of intra-oral exposure, discoloration of the material occurs and mainly affects the luminance parameters L* and the a*, b* scale. As a result, the TP parameters also change [20].

Calculated WI_D_ values ranged between 26.58 and 32.22. Other studies reported WID values ranging from 0.53 to 19.00 units [36].

WI_D_ variations were interpreted according to the perceptibility and acceptability thresholds, in order to assess clinical significance. The perceptibility difference threshold (PT) represents the lower perceptual limit and it can be applicable to study discernible colors by the human visual system. Besides this, differences above the PT are named acceptable differences or color tolerances. These differences are justified by maintaining the differences under an acceptable limit (AT). Studies show that AT and PT values are different. Studies found values for PT and AT of 0.72 and 2.60, respectively [15,19]. Other whiteness thresholds values registered for WI_D_ are 0.70 for no difference, 1.57 for small difference, 2.96 for fairly acceptable and 5.69 for hardly acceptable [36].

The variations in this study after saliva immersion and artificial aging, related to perceptibility and acceptability thresholds, are mostly perceptible, but partially exceed the limit of acceptability. Related to the classification described above, they are fairly acceptable.

Thus, the fourth null hypothesis that color and whiteness changes are above perceptibility thresholds is accepted.

The limitations of the study are related to the types of polymers: only PETG materials were tested in order to make comparisons, which limits the generalizability of the findings. On the other hand, this in vitro study does not simulate real-life usage, and retainers are not only exposed to the oral environment, but also beverages, cleaning solutions, which may affect their properties [61,62]. In addition, the Easyshade clinical device was used, which is generally not recommended for in vitro testing.

In terms of future work, it would be interesting to expand the range of underlying teeth color, which could be different. It would be of high interest to study the cumulative effect of intrinsic and extrinsic factors, including the influence of coloring beverages and cleaning methods, related to the optical properties.

## 5. Conclusions

Within the limitations of this in vitro-conducted study, the following conclusions can be highlighted.

1. Optical properties of PETG clear thermoplastic materials, like TP and OP, increase in a simulated oral environment and the changes become significant after artificial aging. Correlation analyses indicated a migration towards red and yellow.
2. Related to pH values, the optical behavior between the materials is significantly different, but without a more unpropitious environment being evident.
3. In artificial saliva, various commercial PETG materials behave similarly in terms of translucency, and differently related to opalescence. During artificial aging, the tested materials behave significantly differently in terms of optical properties.
4. After the simulated period of 14 days, color changes in some cases even reach the level of extremely marked. Whiteness increases and the differences are mostly perceptible, but partially exceed the limit of acceptability.
5. More studies are needed in terms of extended and multifactorial in vitro and vivo studies of aligners. Providing real conditions for the data obtained in this study may help to understand aesthetic outcomes associated with aligners.

## Figures and Tables

**Table 1:** TP values for the studied materials, in relation to different pH values of artificial saliva (stages I, II, III).

TP	Stage I	Stage II			Stage III		
	Control	0	+	−	0	+	−
L	6.865 ± 0.101	7.136 ± 0.153	7.01 ± 0.156	6.886 ± 0.168	7.204 ± 0.131	7.149 ± 0.117	7.276 ± 0.134
D	6.832 ± 0.148	7.21 ± 0.174	7.243 ± 0.117	7.19 ± 0.176	7.069 ± 0.157	7.203 ± 0.145	7.13 ± 0.101
E	7.192 ± 0.116	7.251 ± 0.187	7.254 ± 0.154	7.308 ± 0.191	7.244 ± 0.112	7.249 ± 0.074	7.301 ± 0.108
C	7.068 ± 0.132	6.63 ± 0.134	6.809 ± 0.152	7.028 ± 0.153	7.177 ± 0.141	7.271 ± 0.146	7.264 ± 0.143

L = Leone, D = Duran, E = Erkodur, C = Cristal, TP = Translucency, (0) = neutral artificial saliva, (+) = basic artificial saliva, (−) = acidic artificial saliva.

**Table 2:** *p*-values (Student *t*-test—unpaired) for the control—stages II and III.

Stage	*p*-Value	L/D	L/E	L/C	D/E	D/C	E/C
control		0.795	0.016	0.126	0.003	0.046	0.255
II	0	0.35	0.171	<0.001	0.637	<0.001	<0.001
	+	0.001	0.948	0.024	<0.001	<0.001	0.014
	−	0.001	<0.001	0.005	0.188	0.435	0.04
III	0	0.146	0.682	0.757	0.073	0.193	0.462
	+	0.52	0.172	0.204	0.405	0.41	0.754
	−	0.544	0.426	0.9	0.695	0.642	0.494

(+) = basic, (−) = acid, (0) = neutral, E = Erkodur, C = Crystal, L = Leone, D = Duran.

**Table 3:** *p*-values (Student *t*-test—paired) for stages II and III.

*p*-Value	Lc/L0	Lc/L+	Lc/L−	Dc/D0	Dc/D+	Dc/D−	Ec/E0	Ec/E+	Ec/E−	Cc/C0	Cc/C+	Cc/C−
contr.-stage II	0.03	0.114	0.883	0.001	<0.001	<0.001	0.536	0.074	0.29	0.005	0.036	0.569
contr.-stage III	0.035	0.078	0.025	0.057	0.005	0.001	0.678	0.475	0.491	0.319	0.04	0.175

(−) = acid, (0) = neutral, (+) = basic, E = Erkodur, C = Crystal, L = Leone, D = Duran.

**Table 4:** Pearson’s correlations and regression tests for groups that experienced significant differences following hydration (stage II) and reconstruction (stage III), respectively, related to TP.

Stage	Material	L*	a*	b*
II	D0	strong, r = 0.716, r^2^ = 0.512 = 51.2%, *p* = 0.733	strong, r = −0.781, r^2^ = 0.609 = 60.9%, *p* = 0.009	weak, r = 0.193, r^2^ = 0.037 = 3.72%, *p* = 0.647
	D+	strong, r = 0.701, r^2^ = 0.49 = 49%, *p* = 0.2	moderate, r = -0.491, r^2^ = 0.24 = 24.01%, *p* = 0.144	moderate, r = −0.546, r^2^ = 0.297 = 29.7%, *p* = 0.007
	D−	very strong, r = 0.850, r^2^ = 0.722 = 72.25%, *p*= 0.379	strong, r = −0.768, r^2^ = 0.588 = 58.8%, *p* = 0.01	very weak, r = 0.162, r^2^ = 0.026 = 2.6%, *p* = 0.017
	C0	very strong, r = 0.924, r^2^ = 0.853 = 85.3%, *p* = 0.920	strong, r = −0.752, r^2^ = 0.564 = 56.4%, *p* = 0.009	strong, r = 0.608, r^2^ = 0.369 = 36.9%, *p* = 0.749
III	D+	very strong, r = 0.826, r^2^ =0.682 = 68.22%, *p* = 0.062	strong, r = −0.772, r^2^ =0.595 = 59.5%, *p* = 0.008	weak, r = 0.379, r^2^ = 0.143 = 14.36%, *p* = 0.017
	D−	weak, r = 0.353, r^2^ = 0.124 = 12.4%, *p* = 0.231	weak, r = 0.201, r^2^ = 0.04 = 4.04%, *p* = 0.595	moderate, r = −0.571, r^2^ = 0.326 32.6%, *p*= 0.001

(+) = basic, (−) = acid, (0) = neutral, L = Leone, D = Duran.

**Table 5:** Mean OP values with standard deviation (SD) for dried control (stage I), hydrated (stage II) and re-dried (stage III) groups are summarized in Table 4 and displayed graphically in Figure 2a,b.

OP	I	II			III		
	Control	0	+	−	0	+	−
L	1.305 ± 0.068	1.352 ± 0.073	1.201 ± 0.07	1.221 ± 0.067	1.387 ± 0.052	1.308 ± 0.086	1.355 ± 0.08
D	1.253 ± 0.07	1.32 ± 0.062	1.245 ± 0.065	1.324 ± 0.05	1.302 ± 0.085	1.293 ± 0.05	1.318 ± 0.038
E	1.294 ± 0.051	1.283 ± 0.031	1.242 ± 0.074	1.216 ± 0.073	1.257 ± 0.097	1.42 ± 0.064	1.377 ± 0.064
C	1.379 ± 0.079	1.428 ± 0.061	1.255 ± 0.056	1.374 ± 0.045	1.425 ± 0.067	1.46 ± 0.043	1.336 ± 0.081

L = Leone, D = Duran, E = Erkodur, C = Cristal, OP = Opalescence, 0 = neutral, + = basic, − = acidic.

**Table 6:** *p*-values (Student *t*-test—unpaired) for stages II and III.

Stage	*p*-Value	L/D	L/E	L/C	D/E	D/C	E/C
control		0.194	0.083	0.336	0.336	0.101	0.061
II	0	0.409	0.018	0.027	0.246	0.006	<0.001
	+	0.021	0.001	0.031	<0.001	0.761	<0.001
	−	0.007	0.907	0.001	0.001	0.146	<0.001
III	0	0.019	0.002	0.197	0.305	0.003	<0.001
	+	0.625	0.005	<0.001	<0.001	<0.001	0.138
	−	0.228	0.527	0.629	0.029	0.551	0.253

(+) = basic, (−) = acid, (0) = neutral, E = Erkodur, C = Crystal, L = Leone, D = Duran.

**Table 7:** *p*-values (Student *t*-test—paired) for the control—stages II and III.

*p*-Value	Lc/L0	Lc/L+	Lc/L−	Dc/D0	Dc/D+	Dc/D−	Ec/E0	Ec/E+	Ec/E−	Cc/C0	Cc/C+	Cc/C−
control-stage II	0.014	0.03	<0.001	0.085	0.989	0.141	0.781	<0.001	0.071	0.188	0.014	0.856
control-stage III	<0.001	<0.001	<0.001	0.283	0.287	0.018	0.496	0.012	0.042	0.236	0.018	0.308

(−) = acid, (0) = neutral, (+) = basic, E = Erkodur, C = Crystal, L = Leone, D = Duran.

**Table 8:** Pearson’s correlations and regression tests for groups that experienced significant differences following hydration (stage II) and reconstruction (stage III), respectively, related to OP.

Stage	Material	a*	b*
II	L−	weak, r = 0.275, r^2^= 0.075 = 7.56%, *p* = 0.573	moderate, r = 0.502, r^2^ =0.25 = 25.5%, *p* = 0.02
	E+	strong, r = 0.623, r^2^ =0.388 = 38.8%, *p* = 0.006	moderate, r = 0.498, r^2^ = 0.24 = 24.8%, *p* < 0.01
III	L0	strong, r = 0.609, r^2^ = 0.371 = 37.1%, *p* = 0.058	weak, r = 0.336, r^2^ =0.113 = 11.3%, *p* = 0.071
	L+	weak, r = 0.377, r^2^ =0.142 = 14.2%, *p* = 0.219	weak, r = 0.274, r^2^ =0.075 = 7.5%, *p* = 0.003
	L−	moderate, r = 0.499, r^2^ =0.249 = 24.9%, *p* = 0.13	weak, r = 0.338, r^2^ =0.114 = 11.4%, *p* = 0.031
	E+	very strong, r = 0.80, r^2^ = 0.64 = 64.3%, *p* = 0.001	very strong, r = 0.8, r^2^ = 0.654 = 65.4%, *p* = 0.003

(−) = acid, (0) = neutral, (+) = basic, E = Erkodur, L = Leone.

**Table 9:** Levels of color changes in stages II and III.

NBS		L	D	E	C
Stage II	0	6.389	6.35	0.895	1.071
	+	2.093	4.719	0.181	4.071
	−	1.518	0.429	2.357	2.338
Stage III	0	3.737	3.396	0.189	3.036
	+	2.635	4.729	4.967	4.978
	−	4.501	6.322	3.204	1.436

C = Crystal, L = Leone, E = Erkodur, D = Duran, (−) = acid, (0) = neutral, (+) = basic. 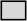 extremely slight change, 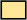 slight change, 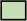 perceivable, 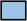 marked change, 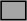 extremely marked change.

**Table 10:** TP values for the studied materials, in relation to different pH values of artificial saliva and after hydrothermal degradation—hydrated (stage IV) and after desiccation (stage V).

TP	I	IV			V		
	Control	0	+	−	0	+	−
L	6.865 ± 0.101	7.065 ± 0.123	7.228 ± 0.131	7.276 ± 0.121	7.348 ± 0.151	7.404 ± 0.147	7.125 ± 0.119
D	6.832 ± 0.148	7.446 ± 0.118	7.537 ± 0.123	7.291 ± 0.131	7.189 ± 0.131	7.235 ± 0.117	7.349 ± 0.123
E	7.192 ± 0.116	7.101 ± 0.084	7.153 ± 0.122	7.284 ± 0.122	7.321 ± 0.095	7.315 ± 0.151	7.312 ± 0.092
C	7.068 ± 0.132	7.189 ± 0.108	7.312 ± 0.133	7.081 ± 0.117	7.618 ± 0.084	7.328 ± 0.132	7.334 ± 0.128

L = Leone, D = Duran, C = Cristal, E = Erkodur, TP = Translucency, (0) = neutral, (+) = basic, (−) = acidic.

**Table 11:** *p*-values (Student *t*-test—unpaired) for the control, hydration and re-dried stages after thermal cycling (stages IV and V).

Stage	*p*-Value	L/D	L/E	L/C	D/E	D/C	E/C
control		0.795	0.016	0.126	0.003	0.046	0.255
IV	0	<0.001	0.565	0.072	<0.001	<0.001	0.07
	+	<0.001	0.344	0.305	<0.001	0.002	0.02
	−	0.804	0.905	0.007	0.918	0.011	0.004
V	0	0.059	0.663	<0.001	0.068	<0.001	<0.001
	+	0.04	0.296	0.352	0.229	0.132	0.841
	−	<0.001	<0.001	<0.001	0.587	0.869	0.784

(+) = basic, (−) = acid, (0) = neutral, E = Erkodur, C = Crystal, L = Leone, D = Duran.

**Table 12:** *p*-values (Student *t*-test—paired) for the control—stages IV and V.

*p*-Value	Lc/L0	Lc/L+	Lc/L−	Dc/D0	Dc/D+	Dc/D−	Ec/E0	Ec/E+	Ec/E−	Cc/C0	Cc/C+	Cc/C−
control-IV	0.029	0.038	0.005	<0.001	<0.001	0.002	0.324	0.66	0.15	0.192	0.026	0.899
control-V	0.003	<0.001	<0.001	0.015	0.001	<0.001	0.08	0.193	0.184	0.101	0.02	0.058

(−) = acid, (0) = neutral, (+) = basic, E = Erkodur, C = Crystal, L = Leone, D = Duran.

**Table 13:** Pearson’s correlations and regression tests for groups that experienced significant differences following thermal cycling, hydration and reconstruction, respectively (stages IV and V).

Stage	Material	L*	a*	b*
IV	L−	strong, r = 0.725, r^2^ = 0.525 = 52.5%, *p* = 0.405	weak, r = 0.376, r^2^ = 0.142 = 14.2%, *p* = 0.306	very weak, r = 0.024, r^2^ = 0.001 = 0.1%, *p* = 0.213
	D0	very strong, r = 0.918, r^2^ = 0.842 = 84.2%, *p* = 0.113	moderate, r = 0.591, r^2^ = 0.349 = 34.9%, *p* = 0.087	moderate, r = 0.430, r^2^ = 0.185 = 18.5%, *p* = 0.732
	D+	very strong, r = 0.955, r^2^ = 0.912 = 91.2%, *p* = 0.021	moderate, r = 0.544, r^2^ = 0.296 = 29.5%, *p* = 0.110	moderate, r = 0.586, r^2^ = 0.344 = 34.36%, *p* = 0.980
	D−	very strong, r = 0.905, r^2^ = 0.819 = 81.9%, *p* = 0.049	strong, r = 0.717, r^2^ = 0.514 = 51.4%, *p* = 0.025	strong, r = 0.659, r^2^ = 0.434 = 43.4%, *p* = 0.715
V	L0	very strong, r = 0.875, r^2^ = 0.765 = 76.5%, *p* = 0.103	strong, r = 0.708, r^2^ = 0.609 =60.9%, *p* = 0.008	weak, r = 0.368, r^2^ = 0.136 = 13.6%, *p* = 0.911
	L+	very strong, r = 0.963, r^2^ = 0.928 = 92.7%, *p* = 0.003	strong, r = 0.812, r^2^ = 0.660 = 66%, *p* = 0.004	very weak, r = 0.182, r^2^ = 0.033 = 3.33%, *p* = 0.824
	L−	very weak, r = 0.115, r^2^ = 0.013 = 1.32%, *p* = 0.002	weak, r = 0.226, r^2^ = 0.051 = 5.12%, *p* = 0.396	weak, r = 0.221, r^2^ = 0.049 = 4.87, *p* = 0.034
	D0	very strong, r = 0.940, r^2^ = 0.883 = 88.3%, *p* = 0.022	strong, r = 0.770, r^2^ = 0.593 = 59.2%, *p* = 0.008	weak, r = 0.278, r^2^ = 0.077 = 7.72%, *p* = 0.068
	D+	strong, r = 0.792, r^2^ = 0.627 = 62.7%, *p* = 0.618	moderate, r = 0.414, r^2^ = 0.172 = 17.1%, *p* = 0.254	moderate, r = 0.408, r^2^ = 0.166 = 0.17%, *p* = 0.007
	D-	very strong, r = 0.841, r^2^ = 0.707 = 70.6%, *p* = 0.244	moderate, r = 0.445, r^2^ = 0.198 = 19.8%, *p* = 0.211	very weak, r = 0.005, r^2^ = 0.0002 = 0.02%, *p* = 0.076

(−) = acid, (0) = neutral, (+) = basic, L = Leone, D = Duran.

**Table 14:** OP values for the studied materials, in relation to different pH environment of artificial saliva, after thermal cycling (stages IV and V).

OP	I	IV			V		
	Control	0	+	−	0	+	−
L	1.305 ± 0.068	1.368 ± 0.067	1.362 ± 0.085	1.424 ± 0.099	1.131 ± 0.106	1.353 ± 0.104	1.361 ± 0.109
D	1.253 ± 0.07	1.42 ± 0.084	1.381 ± 0.114	1.488 ± 0.086	1.169 ± 0.085	1.471 ± 0.037	1.413 ± 0.071
E	1.294 ± 0.051	1.529 ± 0.072	1.477 ± 0.069	1.585 ± 0.069	1.247 ± 0.077	1.407 ± 0.07	1.326 ± 0.055
C	1.379 ± 0.079	1.573 ± 0.081	1.466 ± 0.073	1.628 ± 0.079	1.347 ± 0.047	1.42 ± 0.053	1.383 ± 0.042

(−) = acid, (0) = neutral, (+) = basic, E = Erkodur, C = Crystal, L = Leone, D = Duran.

**Table 15:** *p*-values (Student *t*-test—unpaired) for the stages after thermocycling: hydrated (IV) and re-dried (V).

Stage	*p*-Value	L/D	L/E	L/C	D/E	D/C	E/C
control		0.194	0.807	0.083	0.336	0.101	0.061
IV	0	0.167	<0.001	<0.001	0.008	<0.001	0.104
	+	0.022	0.045	0.004	<0.001	0.736	0.013
	−	0.157	<0.001	<0.001	0.006	<0.001	0.044
V	0	0.191	0.015	0.007	<0.001	<0.001	0.991
	+	0.004	0.212	<0.001	0.026	0.035	0.001
	−	0.214	0.396	0.474	0.008	0.008	0.769

(+) = basic, (−) = acid, (0) = neutral, E = Erkodur, C = Crystal, L = Leone, D = Duran.

**Table 16:** Pearson’s correlations and regression tests for groups that experienced significant differences following artificial aging after hydration and reconstruction, respectively (stages IV and V).

Stage	Material	a*	b*
IV	L0	weak, r = 0.219, r^2^ = 0.048 = 4.8%, *p* = 0.543	very weak, r = 0.047, r^2^ = 0.002 = 0.22%, *p* = 0.13
	L+	moderate, r = 0.423, r^2^ = 0.179 = 17.8%, *p* = 0.21	strong, r = 0.677, r^2^ = 0.459 = 45.9%, *p* = 0.456
	L−	very weak, r = 0.193, r^2^ = 0.037 = 3.7%, *p* = 0.725	very weak, r = 0.039, r^2^ = 0.002 = 0.2%, *p* = 0.03
	D0	very weak, r = 0.047, r^2^ = 0.002 = 0.22%, *p* = 0.62	moderate, r = 0.594, r^2^ = 0.353 = 35.3%, *p* = 0.07
	D+	very weak, r = 0.04, r^2^ = 0.001 = 0.16%, *p* = 0.8	weak, r = 0.206, r^2^ = 0.042 = 4.26%, *p* < 0.001
	D−	strong, r = 0.66, r^2^ = 0.436 = 43.6%, *p* = 0.066	very strong, r = 0.83, r ^2^ = 0.692 = 69.2%, *p* = 0.04
	E0	very weak, r = 0.172, r^2^ = 0.029 = 2.94%, *p* = 0.64	moderate, r = 0.471, r^2^ = 0.221 = 22.1%, *p* > 0.011
	E+	very weak, r = 0.13, r^2^ = 0.017 = 1.71%, *p* = 0.75	weak, r = 0.221, r^2^ = 0.049 = 4.9%, *p* = 0.02
	E−	weak, r = 0.242, r^2^ = 0.059 = 5.87%, *p* = 0.448	moderate, r = 0.512, r^2^ = 0.262 = 26.1%, *p* = 0.73
	C0	moderate, r = 0.542, r^2^ = 0.274 = 27.4%, *p* = 0.12	very weak, r = 0.103, r^2^ = 0.011 = 1.1%, *p* = 0.348
	C+	very weak, r = 0.198, r^2^ = 0.039 = 3.93%, *p* = 0.59	weak, r = 0.39, r^2^ = 0.157 = 15.73%, *p* = 0.393
	C−	very weak, r = 0.242, r^2^ = 0.059 = 5.87%, *p* = 0.44	moderate, r = 0.512,r^2^ = 0.262 = 26.19%, *p* = 0.73
V	L0	weak, r = 0.180, r^2^ =0.032 = 3.22%, *p* = 0.605	strong, r = 0.697, r^2^ =0.486 = 48.9%, *p* = 0.004
	L+	strong, r = 0.605, r^2^ = 0.366 = 36.6%, *p* = 0.062	very weak, r = 0.11,r^2^ = 0.013 = 1.34%, *p* = 0.038
	L−	moderate, r = 0.583, r^2^ = 0.34 = 34%, *p* = 0.049	very weak, r = 0.152, r^2^ = 0.023 = 2.3%, *p* = 0.02
	D0	weak, r = 0.260, r^2^ = 0.067 = 6.74%, *p* = 0.58	strong, r = 0.755, r^2^ = 0.570 = 57%, *p* = 0.057
	D+	weak, r = 0.202, r^2^ = 0.041 = 4.09%, *p* = 0.63	moderate, r = 0.414, r^2^ = 0.171 = 17.1%, *p* = 0.03
	D−	weak, r = 0.371, r^2^ = 0.138 = 13.8%, *p* = 0.33	very strong, r = 0.80, r^2^ = 0.648 = 64.7%, *p* = 0.01

(−) = acid, (0) = neutral, (+) = basic, E = Erkodur, C = Crystal, L = Leone, D = Duran.

**Table 17:** Levels of color changes after thermocycling, stages IV and V.

NBS		L	D	E	C
Stage IV	0	2.55	8	2.07	4.35
	+	3.7	7.61	4.49	2.55
	−	3.99	10.08	7.55	6.32
Stage V	0	3.49	1.63	2.92	2.9
	+	4.37	9.4	2.92	4.81
	−	4.05	7.8	1.73	0.88

C = Crystal, L = Leone, E = Erkodur, D = Duran, (−) = acid, (0) = neutral, (+) = basic. 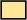 slight change, 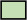 perceivable, 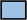 marked change, 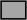 extremely marked change.

**Table 18:** WI_D_ values for all groups.

WI_D_	pH	L	D	E	C
Stage I	control	26.79	26.58	28.33	28.69
Stage II	0	31.00	30.54	28.65	27.98
	+	28.03	28.76	27.91	26.74
	−	25.59	30.52	29.78	30.96
Stage III	0	28.67	28.21	28.12	30.37
	+	28.00	28.66	30.76	31.69
	−	29.06	29.97	29.89	29.51
Stage IV	0	28.18	30.93	28.85	30.88
	+	28.61	30.50	30.52	30.31
	−	29.03	32.01	32.22	32.07
Stage V	0	28.66	26.92	31.42	30.08
	+	28.83	31.70	29.21	31.39
	−	28.77	30.51	28.94	29.08

WI_D_ = whiteness index in dentistry, (0) = neutral, (−) = acidic, (+) = basic, C = Crystal, E = Erkodur, D = Duran, L = Leone.

**Table 19:** ΔWI_D_ differences between the values obtained in different stages and the control group: above PT (perceivable threshold), above AT (acceptable threshold).

WI_D_ Differences	II—I	III—I	IV—I	V—I
L0—Lc	4.21	1.88	1.42	1.87
D0—Dc	3.96	1.63	2.35	0.34
E0—Ec	0.33	−0.21	0.52	3.09
C0—Cc	−0.71	1.68	2.19	1.39
L+)—Lc	1.24	1.21	1.82	2.04
D+)—Dc	2.18	2.08	3.92	5.12
E+)—Ec	−0.42	2.43	2.19	0.88
C+)—Cc	−1.95	3.00	1.62	2.70
L−)—Lc	−1.20	1.27	2.24	1.98
D−)—Dc	3.94	3.39	5.43	3.93
E−)—Ec	1.45	1.56	3.89	0.61
C−)—Cc	2.27	0.82	3.38	0.39

c = control, (0) = neutral, (−) = acidic, (+) = basic, C = Crystal, E = Erkodur, D = Duran, L = Leone. The gray color was introduced to highlight perceivable and acceptable differences.

## Data Availability

The original contributions presented in the study are included in the article; further inquiries can be directed to the corresponding authors.
